# Emotion regulation and heart rate variability may identify the optimal posttraumatic stress disorder treatment: analyses from a randomized controlled trial

**DOI:** 10.3389/fpsyt.2024.1331569

**Published:** 2024-02-08

**Authors:** Danielle C. Mathersul, Jamie M. Zeitzer, R. Jay Schulz-Heik, Timothy J. Avery, Peter J. Bayley

**Affiliations:** ^1^School of Psychology, Murdoch University, Murdoch, WA, Australia; ^2^Centre for Molecular Medicine and Innovative Therapeutics, Health Futures Institute, Murdoch University, Murdoch, WA, Australia; ^3^War Related Illness and Injury Study Center (WRIISC), Veterans Affairs Palo Alto Health Care System, Palo Alto, CA, United States; ^4^Department of Psychiatry and Behavioral Sciences, Stanford University School of Medicine, Stanford, CA, United States; ^5^Mental Illness Research, Education and Clinical Center (MIRECC), Veterans Affairs Palo Alto Health Care System, Palo Alto, CA, United States

**Keywords:** emotion regulation, heart rate variability, posttraumatic stress disorder, yoga, cognitive processing therapy, moderator, predictor, precision medicine

## Abstract

**Introduction:**

High variability in response and retention rates for posttraumatic stress disorder (PTSD) treatment highlights the need to identify "personalized" or "precision" medicine factors that can inform optimal intervention selection before an individual commences treatment. In secondary analyses from a non-inferiority randomized controlled trial, behavioral and physiological emotion regulation were examined as non-specific predictors (that identify which individuals are more likely to respond to treatment, regardless of treatment type) and treatment moderators (that identify which treatment works best for whom) of PTSD outcome.

**Methods:**

There were 85 US Veterans with clinically significant PTSD symptoms randomized to 6 weeks of either cognitive processing therapy (CPT; n = 44) or a breathing-based yoga practice (Sudarshan kriya yoga; SKY; n = 41). Baseline self-reported emotion regulation (Difficulties in Emotion Regulation Scale) and heart rate variability (HRV) were assessed prior to treatment, and self-reported PTSD symptoms were assessed at baseline, end-of-treatment, 1-month follow-up, and 1-year follow-up.

**Results:**

Greater baseline deficit in self-reported emotional awareness (similar to alexithymia) predicted better overall PTSD improvement in both the short- and long-term, following either CPT or SKY. High self-reported levels of emotional response non-acceptance were associated with better PTSD treatment response with CPT than with SKY. However, all significant HRV indices were stronger moderators than all self-reported emotion regulation scales, both in the short- and long-term. Veterans with lower baseline HRV had better PTSD treatment response with SKY, whereas Veterans with higher or average-to-high baseline HRV had better PTSD treatment response with CPT.

**Conclusions:**

To our knowledge, this is the first study to examine both self-reported emotion regulation and HRV, within the same study, as both non-specific predictors and moderators of PTSD treatment outcome. Veterans with poorer autonomic regulation prior to treatment had better PTSD outcome with a yoga-based intervention, whereas those with better autonomic regulation did better with a trauma-focused psychological therapy. Findings show potential for the use of HRV in clinical practice to personalize PTSD treatment.

**Clinical trial registration:**

ClinicalTrials.gov identifier, NCT02366403

## Introduction

Posttraumatic stress disorder (PTSD) is a debilitating mental health condition that can develop following exposure to a traumatic event (1). The efficacy of several trauma-focused therapies for PTSD is well-established (2), across cognitive processing therapy (CPT), prolonged exposure therapy, imaginal exposure, eye-movement desensitization and reprocessing, and trauma-focused cognitive behavioral therapy (CBT) (3–8). Yet, there is significant variability in PTSD treatment response (as well as what denotes response; 9): systematic reviews and meta-analyses of randomized controlled trials (RCTs) suggest that clinically meaningful symptom reduction is achieved in 49%–70% of individuals whereas 60%–72% still retain a PTSD diagnosis posttreatment (10) and anywhere from 0% to 50% are classified as non-responders (11). Furthermore, treatment dropout can be as high as 50% (11) or 60% (2) and is significantly higher among military/defense personnel who have experienced military trauma, especially for trauma-focused therapy (12).

Variability in PTSD treatment response and retention highlights the need to identify “personalized” or “precision” medicine factors that can identify the most optimal intervention before an individual commences treatment. These factors can be considered treatment moderators, which are pre-intervention (baseline) characteristics that alter the treatment effect and identify for whom an intervention works (or does not) (13, 14). Emotion regulation (ER)—the awareness and understanding of, and adaptive responding to, emotional experiences (15–18)—is a promising candidate as a treatment moderator. ER is a transdiagnostic process underlying several emotional-based mental health disorders like PTSD (16, 19) and improves significantly with CBT for these disorders (18). While PTSD is sometimes underrepresented in these systematic reviews and meta-analyses, we recently confirmed that self-reported ER improves with CPT for PTSD (20). Yet, few studies have examined if ER acts as a treatment moderator. One study failed to confirm that ER predicts CBT outcome for anxiety disorders (21). Another recent study of treatment-seeking adults with transdiagnostic emotional symptoms (diagnostic breakdown unavailable) found that higher self-reported use of maladaptive ER strategies at baseline significantly predicted greater improvement in symptoms of anxiety and depression following CBT (22). Yet, these effects are more akin to those of a *non-specific predictor*: a baseline factor that determines which individuals are more likely to respond to treatment, regardless of treatment type (13). In contrast, true *treatment moderators* more precisely identify *which* treatment works best *for whom* (13, 14). To our knowledge, only one study has demonstrated ER acting as a true treatment moderator: for individuals with co-occurring PTSD and substance use disorder (SUD), those who had *more* overall self-reported ER difficulties at baseline had better PTSD outcome with a combined CBT for PTSD plus CBT for SUD, whereas those who had *fewer* baseline ER difficulties had better SUD outcome with CBT for SUD only (23). Further research is clearly needed.

As self-report measures are associated with measurement bias (24, 25), there is value in supplementing self-reported ER with objective biomarkers of ER. One biomarker that is relatively quick, easy, and inexpensive—and could therefore be translatable to clinical practice—is heart rate variability (HRV). HRV is a measure of the differences in the time interval between adjacent heart beats (26, 27) and is considered a well-validated biomarker of ER in both healthy (28, 29) and clinical populations (30), including PTSD (31, 32). Generally, lower HRV is associated with difficulties in ER and poorer mental and physical health while higher HRV is associated with more adaptive regulation and healthier functioning overall (28–30, 33, 34). Two adult studies found that baseline HRV was a significant non-specific predictor of CBT outcome, although the direction of effects diverged. Davies et al. (35) found that low-to-average baseline HRV at rest predicted better anxiety symptom outcome with either CBT or acceptance and commitment therapy for a mixed sample of individuals with DSM-IV anxiety disorders (predominantly panic with or without agoraphobia; only 3.3% PTSD). In contrast, for individuals with primary SUD plus co-occurring PTSD, Soder et al. (36) found that high baseline HRV at rest predicted better PTSD symptom outcome with either CBT for SUD or combined CBT for SUD plus CPT for PTSD. Crucially, neither study found that HRV was able to differentially predict outcome between the two active treatments (i.e., no true treatment moderator effect for HRV), possibly because they were too similar (all CBT-like interventions). In contrast, in a study comparing CBT with yoga for pain, we found that Veterans with Gulf War illness who had higher HRV at baseline experienced greater reductions in pain with yoga, whereas those who had lower baseline HRV experienced greater pain reductions with *CBT* (37). Thus, HRV may be a treatment moderator, depending on the diagnostic profile of individuals or the nature of the two treatments.

Identifying treatment moderators is particularly useful when comparing two or more efficacious interventions, as it could help guide treatment allocation or selection. Yoga is a novel intervention that is garnering an increasing evidence base for its efficacy for mental health disorders, including PTSD (38). In a recently completed non-inferiority RCT, we demonstrated that a breathing-based yoga practice (Sudarshan kriya yoga; SKY) was not clinically inferior to evidence-based CPT at end-of-treatment for symptoms of PTSD, depression, and negative affect among US Veterans (39). In secondary analyses of this RCT, we demonstrated that both self-reported ER and HRV improved with SKY, but only self-reported ER reliably improved with CPT (20).

Here, we aimed to explore if ER (the self-reported Difficulties in Emotion Regulation Scale; DERS) and HRV (5-min during sleep) were *non-specific predictors* or *treatment moderators* of PTSD treatment outcome (measured by the Posttraumatic Stress Disorder Checklist-Civilian Version; PCL-C) among Veterans who received either SKY or CPT. For *Hypothesis A* (*non-specific predictors*), based on extant findings, we expected that poorer overall ER at baseline would predict better PTSD outcome overall, regardless of whether Veterans received SKY or CPT. We similarly expected that HRV would be a non-specific predictor of outcome, although due to inconsistencies in extant literature, we refrained from hypothesizing a direction. For *Hypothesis B* (*treatment moderators*), extant findings hint that *poorer* overall ER at baseline and *lower* baseline HRV may predict better PTSD symptom outcome with *CPT*, whereas *higher* HRV at baseline may predict better PTSD outcome with *SKY*. However, these hypothesized directions are tentative due to divergent findings and a paucity of ER/HRV treatment moderator findings in PTSD populations and/or with yoga-based interventions.

## Materials and methods

### Participants

Participants were US Veterans recruited from the San Francisco Bay Area via advertisements, flyers, social media, etc. All participants had clinically significant PTSD symptoms (≥38 on the PTSD Checklist for DSM-5; PCL-5; 40) and were randomized into the preregistered non-inferiority RCT “Breathing Meditation Intervention for Post-Traumatic Stress Disorder” (ClinicalTrials.gov NCT02366403; 39, 41). Here, we report secondary intent-to-treat (ITT; N = 85) analyses on the DERS and HRV data collected at baseline as non-specific predictors and treatment moderators of PTSD symptom outcome at end-of-treatment and across follow-up (1-month, 1-year). Approximately 25% of baseline cardiac data were excluded due to either equipment failure, missing (i.e., participant did not complete), or poor data quality (20). There were no significant differences between treatment groups in percentage of excluded data or demographics (all *p* >.05) and no significant differences in DERS scores for those with or without cardiac data (all *p* >.29). Groups did not differ on basic demographics (all *p* >.05; **Table 1**) nor on DERS/HRV at baseline (20).

**Table 1:** Baseline demographics and clinical characteristics by treatment group across samples.

	ITT		Valid HRV	
	*CPT (n = 44)*	*SKY (n = 41)*	*CPT (n = 33)*	*SKY (n = 30)*
Age	56.4 (12.9)	57.4 (12.6)	55.4 (14.0)	57.8 (12.4)
% male/female	93.2/6.8	82.9/17.1	90.9/9.1	83.3/16.7
% white	65.9	53.7	63.6	56.7
% married or domestic partner	45.4	34.1	42.5	40.0
% bachelor’s degree or higher	31.8	24.4	36.4	20.0
Total CAPS-5	34.1 (14.4)	32.3 (14.2)	33.8 (15.6)	32.3 (15.3)
Total PCL-C	56.2 (11.7)	56.9 (13.6)	56.2 (12.1)	54.4 (13.6)

ITT, intent-to-treat; HRV, heart rate variability; CPT, cognitive processing therapy; SKY, Sudarshan kriya yoga; CAPS-5, Clinician Administered PTSD Scale for DSM-5; PCL-C, PTSD Checklist – Civilian Version. Except where indicated by %, values are presented in the format M (SD), where M, mean; SD, standard deviation. There were no significant differences between treatment groups on demographics or clinical characteristics at baseline. Full demographics by group are presented in the primary outcomes manuscript (39), and baseline DERS/HRV by group is presented in a secondary analysis manuscript (20).

### Procedure

The protocol was approved by the Stanford University Institutional Review Board and conducted in accordance with the Declaration of Helsinki. The full procedure and primary outcomes of the RCT are described elsewhere (39, 41). Briefly, participants were randomized to receive either CPT or SKY across a 6-week period. Each intervention was delivered according to recommendations and manualized protocols (including home practice on non-class days): CPT was provided as individual, twice-weekly sessions, and SKY was provided as a 5-day group workshop followed by 10, twice-weekly group sessions (39, 41). Multiple clinician-administered, self-reported, and physiological measures were administered at multiple timepoints. Here, we report on the DERS and HRV collected at baseline and analyzed as moderators of treatment outcome for the primary outcome measure of self-reported PTSD symptoms (PCL-C), collected at baseline, end-of-treatment, 1-month follow-up, and 1-year follow-up.

### Measures

#### Posttraumatic Stress Disorder Checklist-Civilian Version

The PCL-C (42) is a 17-item self-report measure that assesses current PTSD symptom severity corresponding to the DSM-IV diagnostic criteria for PTSD (43). Items are rated on a 5-point Likert scale according to how much the symptom bothered the respondent over the past month (1 = “not at all” to 5 = “extremely”), with scores of 38 or higher denoting clinically significant severity levels. The PCL-C has high internal consistency (α = .91–.94), test–retest reliability (r = .66–.68), and convergent validity (r = .93) (44–47). At the time of commencing the primary outcomes study, the psychometric properties of the newer PCL-5 (corresponding to the DSM-5 diagnostic criteria for PTSD (1)) were unknown and no established margin of clinically meaningful change or non-inferiority margin existed. For those reasons, the now replaced PCL-C was the primary outcome measure for the non-inferiority RCT (39, 41) and was our treatment outcome measure for these secondary moderator analyses.

#### The Difficulties in Emotion Regulation Scale

The DERS (15) is a 36-item self-report measure that assesses difficulties in emotional awareness, acceptance, comprehension, and adaptive reactions to emotional experiences. Items are rated on a 5-point Likert scale (0 = “almost never” to 5 = “almost always”), where higher scores reflect more difficulties with ER. The DERS can be scored as a measure of overall difficulties in ER (DERS-Total), as well as six separate subscales (DERS-Non-Acceptance, DERS-Goals, DERS-Impulse, DERS-Awareness, DERS-Strategies, and DERS-Clarity), all of which demonstrate high internal consistency (Cronbach’s *α* = .80–.93) and good test–retest reliability (*ρ_I_* = .57–.89). As some researchers suggest ER should be measured as specific strategy use alongside overall difficulties (e.g., see 48, 49)—and to maintain consistency with our study exploring ER as an outcome of CPT/SKY for PTSD (20)—here, we assessed the DERS-Total and all DERS subscales for the non-specific predictor and treatment moderator analyses.

#### Heart Rate Variability

Ambulatory cardiac data were collected continuously over a 24-h period using Actiwave Cardio monitors (CamNtech Ltd): lightweight, waterproof, chest-worn devices that record heart rate (bpm) and inter-beat-interval (IBI). Data were visually inspected for artifacts (26) and preprocessed and extracted (blind to treatment group) via Kubios HRV Premium 3.1.1 (Kubios, 2019; 50–53), which can calculate HRV from Actiwave data without the need to concurrently measure respiratory rate. We extracted HRV indices from a 5-min epoch of clean, artifact-free data during the first hour of sleep; based on suggestions, this timeframe has the greatest discriminatory power across different mental health disorders (54, 55) and between Veterans and non-Veterans (56, 57). Sleep onset was defined using concurrently recorded triaxial accelerometer (actigraphy) data (Motionlogger, Ambulatory Monitoring, Ardsley NY) and validated algorithms (58) embedded in manufacturer-provided software (ActionW, Ambulatory Monitoring, Ardsley NY).

Per recommendations (26), we extracted two indices each of time-domain HRV (square root of the mean squared differences between successive R–R intervals [RMSSD (ms)], standard deviation of the IBI of normal sinus beats [SDNN (ms)]) and frequency-domain HRV (high-frequency power [HF-HFV (normalized [FFT n.u.], absolute [FFT ms²])]). These are also the most commonly examined indices in PTSD (20, 59, 60) and non-specific predictor/treatment moderator studies (35–37). RMSSD and SDNN are proposed to reflect parasympathetic activation and general autonomic function, with higher values denoting healthier functioning (26, 27, 61). While both normalized and absolute HF-HRV are also proposed to reflect parasympathetic activation (56, 57), normalized HF-HRV can also reflect general autonomic balance (26) and does not always align with absolute HF-HRV (37).

### Analyses

All analyses were ITT, blind to treatment group, and conducted using IBM SPSS Statistics 29.0.0.0. We report all significant (*p* <.05) and trend-level (*p* = .05–1.0) effects alongside model fit comparisons to highlight overarching patterns that warrant further research and replication. The smallest values Akaike’s Information Criterion (AIC), Schwarz’s Bayesian Criterion (BIC), and –2 Log Likelihood (–2LL) identify the model of best fit (62). Examination of residual plots confirmed that linear mixed model assumptions were met (62, 63).

We estimated separate linear mixed models for each of the hypothesized predictors/moderators, with the primary outcome measure (PCL-C total) as the dependent variable. We included intercept for participants as random effects and by-participant random slopes for the effect of time. Fixed effects were baseline PTSD (to better equate groups at baseline; 35, 64), group (CPT, SKY; coded −0.5, +0.5 per recommendations; 65, 66), time (baseline, end-of-treatment, 1-month follow-up, 1-year follow-up; coded to reflect actual time [i.e., 0, 2, 3, 14 months] 67), the predictor/moderator (DERS-Total, DERS-Subscales, RMSSD, SDNN, normalized HF-HRV, absolute HF-HRV), and all interactions except those with baseline PTSD (covariate only). Per recommendations, baseline PTSD, time, and the predictors/moderators were mean-centered (65, 66, 68, 69) and outliers (≥ ± 3 SD) were Winsorized and replaced with the next highest value (70). Note that each of the HRV indices had two outliers.

To account for non-linear associations, we also included quadratic (squared) variables of time, the predictors/moderators, and their interactions. Factors can have different moderating effects across the short- versus long-term, for example, due to the passage of time and interference from extraneous factors that may weaken moderation effects. Therefore, consistent with other studies (e.g., 71–73), we estimated separate models for short-term predictors/moderators (i.e., from baseline to end-of-treatment) and long-term predictors/moderators (from baseline to 1-year follow-up, including end-of-treatment and 1-month follow-up).

Interaction effects and figures were probed and plotted using online Excel templates (http://www.jeremydawson.co.uk/slopes.htm; 68, 74). These figures more accurately represent the statistical model of the moderator interactions rather than arbitrary splits by low/medium/high or one-to-one data point plots of interactions (74). As the primary outcomes paper (75) reported the main effects of group and time and the group × time interactions (which also do not correspond to the current hypotheses), those effects are not reported here. *Hypothesis A* (*non-specific predictors*) was tested by the time × moderator interaction effects and *Hypothesis B (treatment moderators)* was tested by the group × time × moderator interaction effects, including all possible quadratic time/moderator interactions.

## Results

### Hypothesis A (non-specific predictors): baseline DERS/HRV will predict PTSD response to treatment, regardless of group

**Figure 1:**
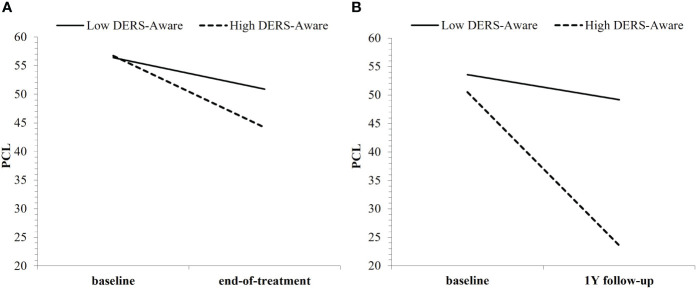
Baseline DERS-Awareness (where “low”/”high” refer to ±1SD from the mean) as a non-specific predictor of both **(A)** short-term and **(B)** long-term PTSD treatment outcome, regardless of group.

We found partial support for *Hypothesis A*: across all DERS and HRV indices, only DERS-Awareness was a non-specific predictor of either short-term (time: *b* = −.12, *t* = −2.52, *p* = .013) or long-term (time: *b* = −.20, *t* = −3.73, *p* <.001; time²: *b* = .02, *t* = 2.28, *p* = .024) PTSD treatment outcome. Consistent with the primary outcomes paper, Veterans showed significant treatment-related reductions in PTSD symptoms following either CPT or SKY; however, the slope of short-term (baseline to end-of-treatment) improvement was steeper for Veterans who had high baseline DERS-Awareness (*p* <.001; i.e., poorer self-reported ER) than for those with low baseline DERS-Awareness (*p* = .008; **Figure 1A**). Across long-term, this effect strengthened, whereby only Veterans who had high DERS-Awareness at baseline (*p* <.001; i.e., poorer self-reported ER) maintained treatment-related reductions in PTSD across follow-up, whereas effects were non-significant (*p* = .301) for those with low DERS-Awareness at baseline (**Figure 1B**).

### Hypothesis B (treatment moderators): baseline DERS/HRV will identify which Veterans respond best to SKY versus CPT for PTSD

#### The Difficulties in Emotion Regulation Scale

**Figure 2:**
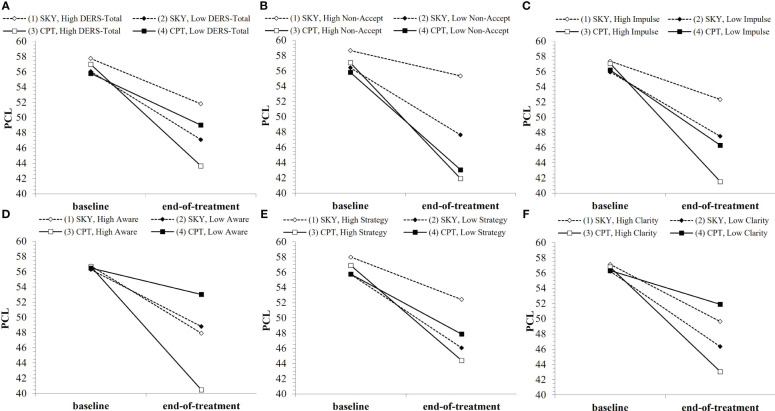
Baseline DERS indices (where “low”/”high” refer to ±1SD from the mean) as moderators of PTSD treatment outcome over the short-term: **(A)** DERS-Total, **(B)** DERS-Non-Acceptance, **(C)** DERS-Impulse, **(D)** DERS-Awareness, **(E)** DERS-Strategy, **(F)** DERS-Clarity. *Note that points are used to differentiate lines rather than to represent data plotting.* Open points (1 and 3) represent high baseline DERS, and enclosed points (2 and 4) represent low baseline DERS. Solid lines/square points represent CPT, and dotted lines/diamond points represent SKY.

The general pattern of findings was for Veterans with higher baseline DERS to have greater reductions in and lower PTSD severity at end-of-treatment with CPT than with SKY (**Figures 2A–F**). This was true for DERS-Total, as well as the subscales DERS-Non-Acceptance, DERS-Impulse, DERS-Awareness, DERS-Strategies, and DERS-Clarity, but not for DERS-Goals, at end-of-treatment (i.e., support for *Hypothesis B* across short-term). Overall, the strongest effects were for the DERS-Non-Acceptance subscale, which was also the only DERS index to consistently moderate outcome across long-term (**Table 2**).

**Table 2:** Final full model for DERS-Non-Acceptance, the DERS index with best fit as moderator of PCL outcome across short-term.

	Est.	SE	95% CI	*t*	*p*
*Fixed effects*					
Intercept	51.99	.79	50.42–53.55	66.02	<.001
Baseline PCL	.85	.05	.74–.95	15.86	<.001
Group	5.04	1.60	1.86–8.22	3.15	.002
Time	−1.66	.27	−2.21 to −1.12	−6.09	<.001
Non-Accept	.19	.10	−.003–.39	1.95	.054
Non-Accept²	.007	.01	−.02–.03	.48	.630
Group*Time	1.31	.55	.23–2.40	2.41	.018
Group*Non-Accept	.38	.17	.04–.72	2.20	.030
Group*Non-Accept²	−.08	.03	−.13 to −.03	−2.96	.004
Time*Non-Accept	.02	.03	−.04–.08	.66	.512
Time*Non-Accept²	.006	.005	−.004–.01	1.20	.234
Group*Time*Non-Accept	.10	.06	−.02–.22	1.70	.093
Group*Time*Non-Accept²	−.02	.009	−.04 to −.002	−2.15	.034
	Est.	SE	Correlation		
*Random effects*					
Time | participant (intercept)	.33	.63	-.74		

DERS, The Difficulties in Emotion Regulation Scale; PCL, PTSD Checklist; Est., estimate; SE, standard error; CI, confidence interval; Non-Accept, baseline DERS Non-Acceptance subscale. Satterthwaites approximations used to calculate p-values; Wald method used to calculate confidence intervals.

For short-term treatment outcome, there was a significant interaction effect for group × time × DERS-Total (*b* = .03, *t* = 2.00, *p* = .049) and trend-level-to-significant effects for group × time × DERS for all subscales except DERS-Goals (DERS-Non-Acceptance: *b* = .10, *t* = 1.70, *p* = .093; DERS-Impulse: *b* = .12, *t* = 1.82, *p* = .072; DERS-Awareness: *b* = .19, *t* = 2.03, *p* = .045; DERS-Strategy: *b* = .10, *t* = 1.76, *p* = .079; DERS-Clarity: *b* = .24, *t* = 2.14, *p* = .035; **Supplementary Table 1**). There was also a significant interaction effect for group × time × DERS-Non-Acceptance² (*b* = −.02, *t* = −2.15, *p* = .034). While all group and baseline DERS-Total combinations showed significant reductions in PTSD with treatment (all *p* <.032), when Veterans had high DERS-Total (i.e., poorer self-reported ER) at baseline, they tended to have greater treatment-related reductions from baseline to end-of-treatment (*p* = .055) and significantly lower PTSD severity at end-of-treatment (*p* =.005) with CPT than with SKY (**Figure 2A**). Except for DERS-Goals, the DERS-subscales tended to show this similar pattern of greater treatment-related reductions from baseline to end-of-treatment (Non-Acceptance: *p* = .005; Impulse: *p* = .026; Awareness: *p* = .076) and significantly lower PTSD severity at end-of-treatment (Non-Acceptance: *p* <.001; Impulse: *p* =.002; Awareness: *p* = .018; Strategy: *p* = .011; Clarity: *p* = .029) for Veterans who had high baseline DERS and received CPT versus SKY (**Figures 2B–F**).

However, these DERS moderator effects were less consistent across long-term follow-up: there were interaction effects for group × time × DERS-Impulse (*b* = .14, *t* = 1.73, *p* = .085), group × time × DERS² (Non-Acceptance: *b* = −.03, *t* = −2.73, *p* = .007; Impulse: *b* = −.02, *t* = −1.84, *p* = .067), and group × time² × DERS-Non-Acceptance² (*b* = .005, *t* = 2.76, *p* = .006; **Supplementary Table 2**). While effects tapered off over long-term follow-up, Veterans who had high or average-to-high baseline DERS[-Non-Acceptance] had greater treatment-related reductions in PTSD severity across follow-up with CPT (**Supplementary Figure 1A**) versus SKY (**Supplementary Figure 1B**).

#### Heart Rate Variability

**Figure 3:**
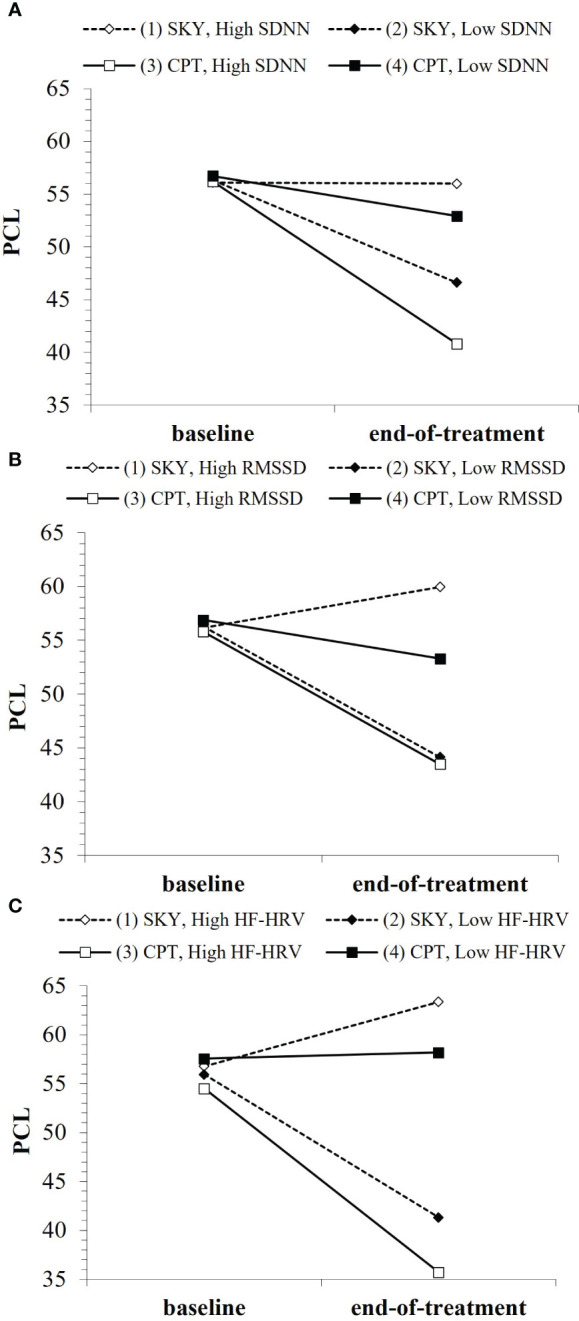
Baseline HRV indices (where “low”/”high” refer to ±1SD from the mean) as moderators of PTSD treatment outcome over the short-term: **(A)** SDNN, **(B)** RMSSD, **(C)** HF-HRV. *Note that points are used to differentiate lines rather than to represent data plotting.* Open points (1 & 3) represent high baseline HRV, enclosed points (2 & 4) represent low baseline HRV. Solid lines/square points represent CPT, dotted lines/diamond points represent SKY.

We found support for *Hypothesis B* for SDNN, RMSSD, and absolute HF-HRV, but not for normalized HF-HRV. The general pattern of findings was for Veterans with lower baseline HRV to have greater treatment-related reductions in PTSD severity with SKY and for Veterans with higher baseline HRV to have greater treatment-related reductions in PTSD severity with CPT (**Figures 3A–C**). Overall, the best model fit was for absolute HF-HRV (**Table 3**), with SDNN and RMSSD almost equivalent to each other. All HRV models were stronger fits than all DERS models (**Supplementary Tables 1–4**).

**Table 3:** Final full model for absolute HF-HRV (ms²), the HRV index with best fit as moderator of PCL outcome across short-term.

	Est.	SE	95% CI	*t*	*p*
*Fixed effects*					
Intercept	52.92	.89	51.14–54.71	59.28	<.001
Baseline PCL	.92	.05	.82–1.01	19.43	<.001
Group	2.85	1.76	−.66–6.36	1.63	.109
Time	−1.09	.30	−1.69 to −.49	−3.65	<.001
HF-HRV	−.0002	.001	−.002–.002	−.15	.884
HF-HRV²	4.18e^−7^	3.55e^−7^	−2.91e^−7^–1.13e^−6^	1.18	.243
Group*Time	.84	.60	−.35–2.04	1.41	.162
Group*HF-HRV	.006	.002	.001–.01	2.64	.011
Group*HF-HRV²	−1.37e^−6^	7.11e^−7^	−2.80e^−6^–4.68e^−8^	−1.93	.058
Time*HF-HRV	3.47e^−5^	.0004	−.0007–.0008	.10	.923
Time*HF-HRV²	1.01e^−7^	1.19e^−7^	−1.37e^−7^–3.40e^−7^	.85	.400
Group*Time*HF-HRV	.002	.0007	.0001–.003	2.21	.031
Group*Time*HF-HRV²	−3.77e^−7^	2.39e^−7^	−8.55e^−7^–1.00e^−7^	−1.58	.120
	Est.	SE	Correlation		
*Random effects*					
Time | participant (intercept)	.13	.59	−.75		

HRV, heart rate variability; PCL, PTSD Checklist; Est., estimate; SE, standard error; CI, confidence interval; HF-HRV, baseline absolute high frequency power HRV (FFT ms²) index. Satterthwaites approximations used to calculate p-values; Wald method used to calculate confidence intervals.

For short-term treatment outcome, there were interaction effects for group × time × HRV (SDNN: *b* = .05, *t* = 2.11, *p* = .039; RMSSD: *b* = .05, *t* = 1.92, *p* = .059; HF-HRV [ms²]: *b* = .002, *t* = 2.21, *p* = .031) and group × time × HRV² (SDNN²: *b* = −.0009, *t* = −2.32, *p* = .023; RMSSD²: *b* = −.0004, *t* = −1.88, *p* = .064; **Supplementary Table 3**). When Veterans had low HRV at baseline, their PTSD only significantly improved (i.e., symptoms only significantly reduced) with SKY (SDNN: *p* = .002; RMSSD: *p* = .002; HF-HRV [ms²]: *p* <.001) and they had significantly lower PTSD severity at end-of-treatment with SKY than with CPT (SDNN: *p* =.046; RMSSD: *p* = .020; HF-HRV [ms²]: *p* =.002; **Figures 3A–C**). In contrast, when Veterans had high HRV at baseline, their PTSD only consistently improved with CPT (SDNN: *p* = .005; RMSSD: *p* = .058; HF-HRV [ms²]: *p* =.097) and they had significantly lower PTSD severity at end-of-treatment with CPT than with SKY (SDNN: *p* =.010; RMSSD: *p* = .016; HF-HRV [ms²]: *p* =.002; **Figures 3A–C**).

Similarly, for long-term follow-up, there were interaction effects for group × time² × HRV (SDNN: *b* = −.010, *t* = −2.02, *p* = .045; HF-HRV [ms²]: *b* = −.0003, *t* = −2.03, *p* = .044) and group × time² × HRV² (SDNN²: *b* = .0002, *t* = 2.14, *p* = .034; RMSSD²: *b* = 8.40e^−5^, *t* = 1.77, *p* = .079; HF-HRV² [ms²]: *b* = 8.54e^−8^, *t* = 1.70, *p* = .092; **Supplementary Table 4**). While effects tapered off over long-term follow-up, Veterans who had low baseline HRV had greater treatment-related reductions in PTSD severity across follow-up with SKY (**Supplementary Figures 2B, D, F**), whereas Veterans who had high or average-to-high baseline HRV had greater treatment-related reductions in PTSD severity across follow-up with CPT (**Supplementary Figures 2A, C, E**).

## Discussion

To our knowledge, this is the first study to examine both self-reported ER and HRV, within the same study, as both non-specific predictors and moderators of PTSD treatment outcome. We found that self-reported ER acted as a non-specific predictor, such that a greater baseline deficit in emotional awareness predicted better overall PTSD improvement across both short- and long-term, regardless of treatment group. Self-reported ER was also a significant treatment moderator: more overall difficulties with ER at baseline—and most strongly for high levels of emotional response non-acceptance—were associated with better PTSD treatment response with CPT than with SKY, across both short- and long-term. However, all significant HRV indices were stronger moderators than all DERS scales. Across SDNN, RMSSD, and absolute (but not normalized) HF-HRV, Veterans with *lower* baseline HRV (poorer autonomic function) had better PTSD treatment response with *SKY*, whereas Veterans with *higher* or *average-to-high* baseline HRV (better autonomic function) had better PTSD treatment response with *CPT*, across both short- and long-term. Absolute HF-HRV was the strongest moderator overall.

Non-specific predictor studies employing self-reported ER are rare and only recently emerging. However, our findings are consistent with the handful of extant studies demonstrating that poorer ER at baseline predicts better overall treatment response among individuals with emotional disorders. This effect occurs for self-reported expressive suppression (22) as well as experimentally induced negative emotion reactivity (76) and behavioral avoidance (35) in CBT-like therapies for transdiagnostic anxiety and/or emotional disorders. Here, we extend these findings to confirm self-reported emotional awareness as a *non-specific predictor* of PTSD outcome with either a CBT-like therapy (CPT) or yoga-based intervention (SKY). Specifically, impairment in emotional awareness—difficulty acknowledging or attending to feelings, also known as alexithymia—was associated with better overall PTSD treatment response. This is perhaps unsurprising given the high prevalence of alexithymia and correlation with symptom severity among PTSD populations (77, 78). Furthermore, to our knowledge, only one study has demonstrated self-reported ER acting as a true *treatment moderator*: among individuals with co-occurring PTSD and SUD, those who had more overall difficulties with ER at baseline had better PTSD outcome with combined CBT for PTSD/SUD, whereas those who had fewer baseline ER difficulties had better SUD outcome with CBT for SUD only (23). Here, we also confirmed that Veterans who had more overall difficulties with ER at baseline (especially greater difficulties accepting distress and a greater tendency for distress to elicit secondary emotions such as guilt or shame) had better PTSD outcome with CPT. Together, these findings suggest that for individuals with PTSD, if they also have poor ER, they are more likely to benefit from a trauma-focused psychological therapy than a non-trauma-focused psychological therapy (23) or yoga-based intervention (SKY; this study). While CBT-based therapies generally target maladaptive ER by promoting emotional awareness and acceptance and increasing adaptive ER strategies (18, 79, 80)—and we have previously demonstrated that both CPT and SKY improve self-reported ER in individuals with PTSD (20)—it is possible that trauma-focused therapies more directly target PTSD-related ER difficulties and therefore were more effective for individuals with poorer ER, whereas individuals with better ER were able to benefit from either CPT or SKY.

There are some important caveats to these conclusions. First, while self-reported ER had a statistical treatment moderator effect, this effect only resulted in a steeper slope for CPT than SKY; it did not suggest that those receiving SKY were treatment non-responders. Second, PTSD treatment dropout is more likely to occur among people with more overall self-reported ER difficulties at baseline (81) and especially for trauma-focused therapy (12). This highlights the importance of identifying predictors of dropout as well as treatment response. Furthermore, there is high heterogeneity in PTSD presentation: not only are there over 600,000 possible symptom combinations to receive a PTSD diagnosis (82), but different trauma types are associated with different symptom cluster patterns (83) that may require different treatment approaches. At the same time, there is much debate over whether ER should be measured as overall difficulties or competence or more specific ER strategy use (e.g., see 48, 49). Add to this the inherent bias in self-report measures (24, 25) and there is a need to expand our measurement of ER, particularly to objective biomarkers like HRV (28–32).

We demonstrated that *lower* baseline HRV was associated with better PTSD treatment response with *SKY*, whereas *higher* or *average-to-high *baseline HRV was associated with better PTSD treatment response with *CPT*. These moderator effects were strongest for absolute HF-HRV though were consistent across SDNN and RMSSD and across both short- and long-term. Importantly, all HRV moderator effects were stronger than the self-reported ER moderator effects. HRV is proposed to reflect autonomic resilience and capacity to adapt to stressors (84), so our findings suggest that Veterans with poorer autonomic regulation had better PTSD outcome with SKY whereas those with better autonomic regulation did better with CPT. This is consistent with our recent study demonstrating that SKY significantly improved HRV whereas CPT did not reliably improve HRV (20): it makes sense that those with the poorest autonomic function would gain the largest benefits from an intervention that directly improves autonomic function. It is also possible that this autonomic dysregulation might affect an individual’s ability to engage effectively in psychological treatment. Among emotional disorders, no extant study has demonstrated HRV as a true moderator. However, it is interesting to note that our findings are consistent with another non-specific predictor study where, among individuals with clinically significant PTSD symptoms (primary SUD), those with high baseline HRV had better PTSD symptom outcome with psychological therapy (36). In contrast, another study found, among individuals with predominantly panic with or without agoraphobia, those with low-to-average baseline HRV had better anxiety symptom outcome with psychological therapy (35). One possible reason for these discrepancies is the clinical characteristics of the sample, particularly the predominance of PTSD symptoms. Another is the HRV measure: our findings are consistent with Soder et al., and both they and we found effects for absolute HF-HRV, whereas Davies et al. found an opposite pattern with normalized HF-HRV (and we found no predictor/moderator effects). We have previously shown that normalized and absolute HF-HRV do not always align (37). Future research could explore if these differences in baseline HRV are associated with different PTSD symptom clusters, for example, individuals with lower baseline HRV might have more severe and/or a higher number of symptoms within the autonomic arousal/reactivity cluster.

The major strength of our study is the investigation of both non-specific predictors and treatment moderators, a practice that is highly recommended yet rarely performed (85, 86). Non-specific predictors are baseline factors that determine which individuals are more likely to respond to treatment, regardless of type (13), whereas true treatment moderators more precisely identify which treatment works best for whom (13, 14). In the primary outcomes study, we demonstrated that SKY was not clinically inferior to evidence-based CPT at end-of-treatment for symptoms of PTSD, depression, and negative affect among US Veterans (39). Thus, identifying treatment moderators is particularly useful when comparing these two efficacious interventions, as it could help guide treatment choice via precision medicine and potentially improve both treatment efficacy and cost effectiveness (85, 86). Another strength is our exploration of both self-reported ER and HRV as predictors and moderators. While self-reported ER was a significant treatment moderator, HRV had the strongest effects and was most clearly able to identify at baseline which Veterans would respond best to either CPT or SKY. Although other objective markers of ER such as neuroimaging and event-related brain potentials show promise as potential targets for precision medicine among emotional disorders (e.g., 87, 88), HRV is significantly faster, easier, and less expensive to use, making it more easily translatable to clinical practice.

Our study has some limitations. First, the sample size is moderate and there is a large amount of missing HRV data, which may explain some of our trend-level effects and why we did not find effects for normalized HF-HRV. Nevertheless, our study is fully powered for the non-inferiority primary outcome analyses, is similar to (if not larger than) the extant ER/HRV moderator studies (23, 35, 36), and demonstrates consistent patterns across measures. As ambulatory methods typically incur large proportions of missing data (e.g., poor signal quality due to issues with motion or skin adherence) (89, 90)—and indeed, our proportion of missing data (25%) is comparable with other ambulatory/sleep HRV studies (91, 92)—this should be considered in design planning of future studies, such as longer assessment durations (1–2 weeks) to maximize opportunities for usable data. Second, our 5-min HRV indices were recorded during sleep, rather than the typical “awake at-rest” condition, and might therefore be more influenced by sleep than wake physiology. Furthermore, as these were ambulatory rather than lab-based assessments, we cannot rule out the possible influence of nighttime substance use (e.g., alcohol, nicotine). However, while replication is recommended, sleep assessment has the advantage of removing cognitive and behavioral influences on physiology (20) and our findings are consistent with those of Soder et al. (36) who examined 5-min awake at-rest. In addition, while self-report findings from our sample (93) suggested an average 66%–69% likelihood of sleep apnea (as measured by the Multivariate Apnea Prediction Index [MAPI]; 94), restless legs syndrome (as measured by the Restless Legs Syndrome Diagnostic Index [RLS-DI]; 95) was absent (ruling out this potential cause of movement artifact) and there were no group differences across subjective self-reported sleep diary or PTSD sleep symptom (insomnia/nightmares) measures (93). Third, our inclusion criterion was clinically significant PTSD symptoms rather than a PTSD diagnosis and as such, not all individuals met diagnostic criteria for PTSD. However, this aligns with clinical practice models and research that focus more on dimensional distress rather than categorical diagnoses, where subthreshold symptoms are often predictive of future diagnosis and warrant intervention (96). Finally, as treatment moderator studies are rare, it is unclear whether our CPT findings generalize to all trauma-focused or psychological therapies or if our SKY findings generalize to all yoga-based or mindfulness-based interventions. This highlights the need for more treatment moderator analyses across various clinical demographics and interventions to improve treatment development, delivery, and outcome (85, 86).

In conclusion, to our knowledge, this is the first study to examine both self-reported ER and HRV, within the same study, as both non-specific predictors and moderators of PTSD treatment outcome. While we found evidence for self-reported ER acting as a non-specific predictor and treatment moderator, all significant HRV indices were stronger moderators than all DERS scales. Across SDNN, RMSSD, and absolute (but not normalized) HF-HRV, Veterans with *lower* baseline HRV (poorer autonomic function) had better PTSD treatment response with *SKY*, whereas Veterans with *higher* or *average-to-high* baseline HRV (better autonomic function) had better PTSD treatment response with *CPT*, across both short- and long-term. Absolute HF-HRV was the strongest moderator overall. Findings show potential for the use of HRV in clinical practice to personalize PTSD treatment.

## Author’s note

A subset of preliminary data from this study were delivered in an oral presentation at the *21^st^ World Congress of International Organization of Psychophysiology* in Geneva, Switzerland in June 2023 and published in abstract form at *International Journal of Psychophysiology, 188(Supp2023)*, 80.

## Data availability statement

The datasets generated and/or analyzed during the current study are not publicly available due to institutional regulations protecting service member data but are available from the corresponding (DCM) or senior (PJB) authors on reasonable written request (may require data use agreements to be developed).

## Ethics statement

The studies involving humans were approved by Stanford University Institutional Review Board. The studies were conducted in accordance with the local legislation and institutional requirements. The participants provided their written informed consent to participate in this study.

## Author contributions

DCM: Conceptualization, Data curation, Formal analysis, Investigation, Methodology, Validation, Visualization, Writing – original draft, Writing – review & editing. JZ: Methodology, Resources, Software, Supervision, Validation, Writing – review & editing. RJS-H: Data curation, Investigation, Writing – review & editing. TJA: Investigation, Visualization, Writing – review & editing. PB: Conceptualization, Funding acquisition, Investigation, Methodology, Project administration, Resources, Supervision, Validation, Writing – review & editing.

## Supplementary material

Supplementary Figure 1 Baseline DERS-Non-Acceptance (where “low”/”high” refer to ±1SD from the mean and “av.” refers to the mean) as a moderator of PTSD outcome with **(A)** CPT and **(B)** SKY, over the long-term.

Supplementary Figure 2 Baseline HRV (where “low”/”high” refer to ±1SD from the mean and “av.” refers to the mean) as moderators of PTSD outcome with (**A**, **C**, **E**; left side) CPT and (**B**, **D**, **F**; right side) SKY, over the long-term: (**A**, **B**; top) SDNN, (**C**, **D**; middle) RMSSD, (**E**, **F**; bottom) HF-HRV.
