# Supplementary material for: Emotion regulation and heart rate variability may identify the optimal posttraumatic stress disorder treatment: analyses from a randomized controlled trial
Source: Front Psychiatry. 2024 Feb 8;15:1331569. doi: 10.3389/fpsyt.2024.1331569 (PMC10881770; doi:10.3389/fpsyt.2024.1331569)
Supplement: Supplementary file 1 [file Table_1.docx]

**Supplementary Table 1**

*Comparison of mixed models of DERS scales as moderators of PCL outcome across short-term*

| Parameter | Total | | **Non-Acceptance** | | Goals | | Impulse | | Awareness | | Strategies | | Clarity | |
| --- | --- | --- | --- | --- | --- | --- | --- | --- | --- | --- | --- | --- | --- | --- |
|  | Est. | SE | **Est.** | **SE** | Est. | SE | Est. | SE | Est. | SE | Est. | SE | Est. | SE |
| *Fixed Effects* |  |  |  |  |  |  |  |  |  |  |  |  |  |  |
| Intercept | 52.27 | .76 | **51.99** | **.79** | 51.93 | .78 | 51.79 | .86 | 52.05 | .72 | 52.15 | .78 | 52.18 | .74 |
| Grp*Time*DERS | .03 | .02 | **.10** | **.06** | .08 | .09 | .12 | .07 | .19 | .09 | .10 | .06 | .24 | .11 |
| Grp*Time*DERS² | -.0001 | .0005 | **-.02** | **.009** | -.01 | .01 | -.01 | .01 | -.008 | .01 | -.001 | .007 | .02 | .02 |
| *Fit Statistics* |  | |  | |  |  |  | |  | |  |  |  |  |
| Parameters | 15 | | **15** | | 15 | | 15 | | 15 | | 15 | | 15 | |
| AIC | 1035.84 | | **1023.52** | | 1038.40 | | 1036.10 | | 1028.98 | | 1035.91 | | 1031.68 | |
| BIC | 1080.90 | | **1068.58** | | 1083.46 | | 1081.16 | | 1074.04 | | 1080.96 | | 1076.73 | |
| LL | 1005.84 | | **993.52** | | 1008.40 | | 1006.10 | | 998.98 | | 1005.91 | | 1001.68 | |

*Note.* DERS = The Difficulties in Emotion Regulation Scale; PCL = PTSD Checklist; Est. = estimate; SE = standard error; Grp = treatment group; AIC = Akaike’s Information Criterion; BIC = Schwarz’s Bayesian Criterion; LL = -2 Log Likelihood. ***Bold font*** *indicates the DERS moderator with the best fit, of which, the full model is displayed in Table 2.*

**Supplementary Table 2**

*Comparison of mixed models of DERS scales as moderators of PCL outcome across long-term*

| Parameter | Total | | **Non-Acceptance** | | Goals | | Impulse | | Awareness | | Strategies | | Clarity | |
| --- | --- | --- | --- | --- | --- | --- | --- | --- | --- | --- | --- | --- | --- | --- |
|  | Est. | SE | **Est.** | **SE** | Est. | SE | Est. | SE | Est. | SE | Est. | SE | Est. | SE |
| *Fixed Effects* |  |  |  |  |  |  |  |  |  |  |  |  |  |  |
| Intercept | 44.70 | 1.83 | **41.74** | **1.82** | 43.69 | 1.81 | 42.14 | 2.01 | 44.22 | 1.72 | 42.98 | 1.88 | 44.28 | 1.74 |
| Grp*Time*DERS | -.0009 | .0006 | **-.03** | **.01** | .05 | .11 | -.02 | .01 | .07 | .11 | .07 | .07 | .04 | .14 |
| Grp*Time*DERS² | 8.12e^-5^ | .0001 | **.005** | **.002** | -.03 | .02 | .003 | .002 | -.03 | .02 | -.007 | .008 | -.02 | .03 |
| *Fit Statistics* |  | |  | |  |  |  | |  | |  |  |  |  |
| Parameters | 21 | | **21** | | 21 | | 21 | | 21 | | 21 | | 21 | |
| AIC | 1951.82 | | **1928.58** | | 1949.54 | | 1048.73 | | 1931.08 | | 1953.48 | | 1950.38 | |
| BIC | 2026.68 | | **2003.44** | | 2024.40 | | 2023.59 | | 2005.93 | | 2028.33 | | 2025.24 | |
| LL | 1909.82 | | **1886.58** | | 1907.54 | | 1906.73 | | 1889.08 | | 1911.48 | | 1908.38 | |

*Note.* DERS = The Difficulties in Emotion Regulation Scale; PCL = PTSD Checklist; Est. = estimate; SE = standard error; Grp = treatment group; AIC = Akaike’s Information Criterion; BIC = Schwarz’s Bayesian Criterion; LL = -2 Log Likelihood. ***Bold font*** *indicates the DERS moderator with the best fit.*

**Supplementary Table 3**

*Comparison of mixed models of HRV indices as moderators of PCL outcome across short-term*

| Parameter | SDNN | | RMSSD | | **HF-HRV (ms²)** | | HF-HRV (n.u.) | |
| --- | --- | --- | --- | --- | --- | --- | --- | --- |
|  | Est. | SE | Est. | SE | **Est.** | **SE** | Est. | SE |
| *Fixed Effects* |  |  |  |  |  |  |  |  |
| Intercept | 52.71 | .87 | 53.25 | .86 | **52.92** | **.89** | 52.90 | .87 |
| Grp*Time*HRV | .05 | .02 | .05 | .03 | **.002** | **.0007** | -.004 | .03 |
| Grp*Time*HRV² | -.001 | .0004 | -.0004 | .0002 | **-3.77e^-7^** | **2.39e^-7^** | -.0007 | .001 |
| *Fit Statistics* |  | |  | |  | |  | |
| Parameters | 15 | | 15 | | **15** | | 15 | |
| AIC | 771.95 | | 772.53 | | **753.70** | | 776.82 | |
| BIC | 812.73 | | 813.31 | | **794.48** | | 817.59 | |
| LL | 741.95 | | 742.53 | | **723.70** | | 746.82 | |

*Note.* HRV = heart rate variability; PCL = PTSD Checklist; SDNN = standard deviation of the IBI of normal sinus beats (ms); RMSSD = square root of the mean squared differences between successive R-R intervals (ms); HF-HRV (ms²) = absolute high frequency power HRV (FFT ms²); HF-HRV (ms²) = normalised high frequency power HRV (FFT n.u.); Est. = estimate; SE = standard error; Grp = treatment group; AIC = Akaike’s Information Criterion; BIC = Schwarz’s Bayesian Criterion; LL = -2 Log Likelihood. ***Bold font*** *indicates the HRV moderator with the best fit, of which, the full model is displayed in Table 3.*

**Supplementary Table 4**

*Comparison of mixed models of HRV indices as moderators of PCL outcome across long-term*

| Parameter | SDNN | | RMSSD | | **HF-HRV (ms²)** | | HF-HRV (n.u.) | |
| --- | --- | --- | --- | --- | --- | --- | --- | --- |
|  | Est. | SE | Est. | SE | **Est.** | **SE** | Est. | SE |
| *Fixed Effects* |  |  |  |  |  |  |  |  |
| Intercept | 45.50 | 2.04 | 46.59 | 1.93 | **45.23** | **2.03** | 43.58 | 2.07 |
| Grp*Time*HRV | .03 | .03 | .03 | .03 | **.001** | **.0009** | -.02 | .03 |
| Grp*Time*HRV² | -.0008 | .0005 | -.0004 | .0003 | **-3.88e^-7^** | **2.90e^-7^** | -4.49e^-5^ | .001 |
| *Fit Statistics* |  | |  | |  | |  | |
| Parameters | 21 | | 21 | | **21** | | 21 | |
| AIC | 1443.57 | | 1445.88 | | **1430.82** | | 1448.64 | |
| BIC | 1512.30 | | 1514.61 | | **1499.55** | | 1517.37 | |
| LL | 1401.57 | | 1403.88 | | **1388.82** | | 1409.64 | |

*Note.* HRV = heart rate variability; PCL = PTSD Checklist; SDNN = standard deviation of the IBI of normal sinus beats (ms); RMSSD = square root of the mean squared differences between successive R-R intervals (ms); HF-HRV (ms²) = absolute high frequency power HRV (FFT ms²); HF-HRV (ms²) = normalised high frequency power HRV (FFT n.u.); Est. = estimate; SE = standard error; Grp = treatment group; AIC = Akaike’s Information Criterion; BIC = Schwarz’s Bayesian Criterion; LL = -2 Log Likelihood. ***Bold font*** *indicates the HRV moderator with the best fit.*
