# Supplementary figures and images for: Emotion regulation and heart rate variability may identify the optimal posttraumatic stress disorder treatment: analyses from a randomized controlled trial
Source: Front Psychiatry. 2024 Feb 8;15:1331569. doi: 10.3389/fpsyt.2024.1331569 (PMC10881770; doi:10.3389/fpsyt.2024.1331569)

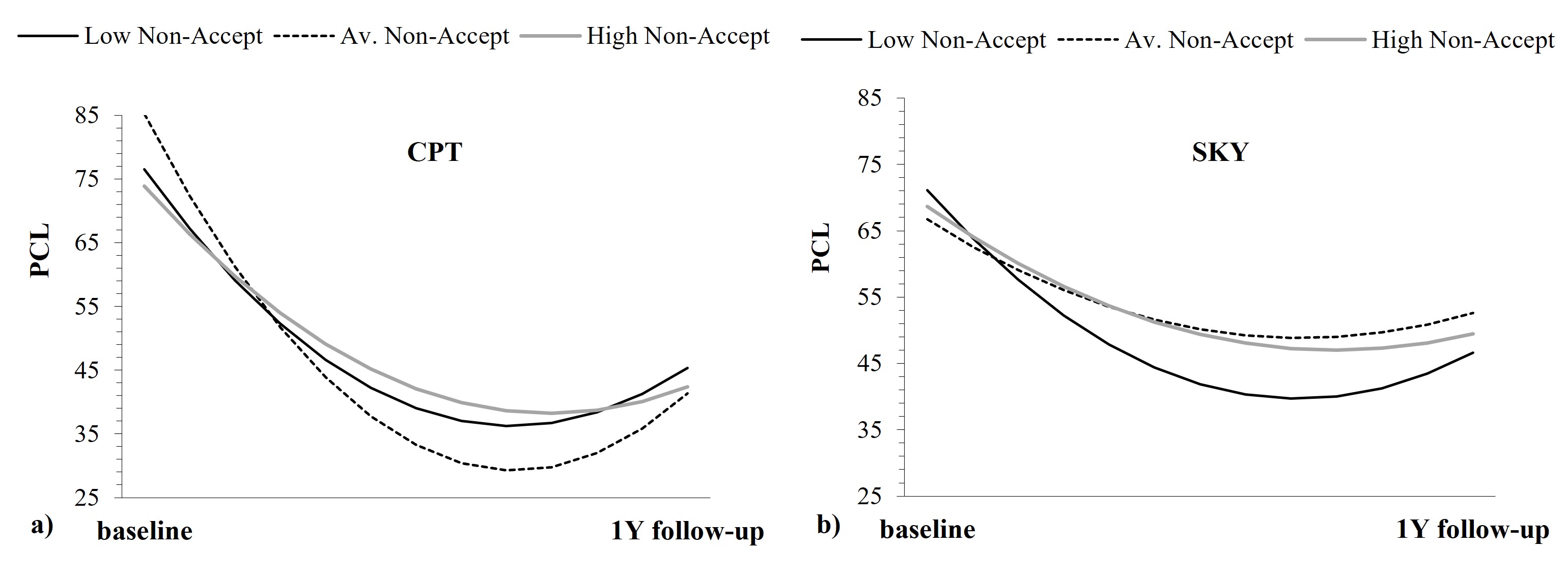

Supplement: Supplementary Figure 1 — Baseline DERS-Non-Acceptance (where “low”/”high” refer to ±1SD from the mean and “av.” refers to the mean) as a moderator of PTSD outcome with (A) CPT and (B) SKY, over the long-term. [file Image_1.jpeg]

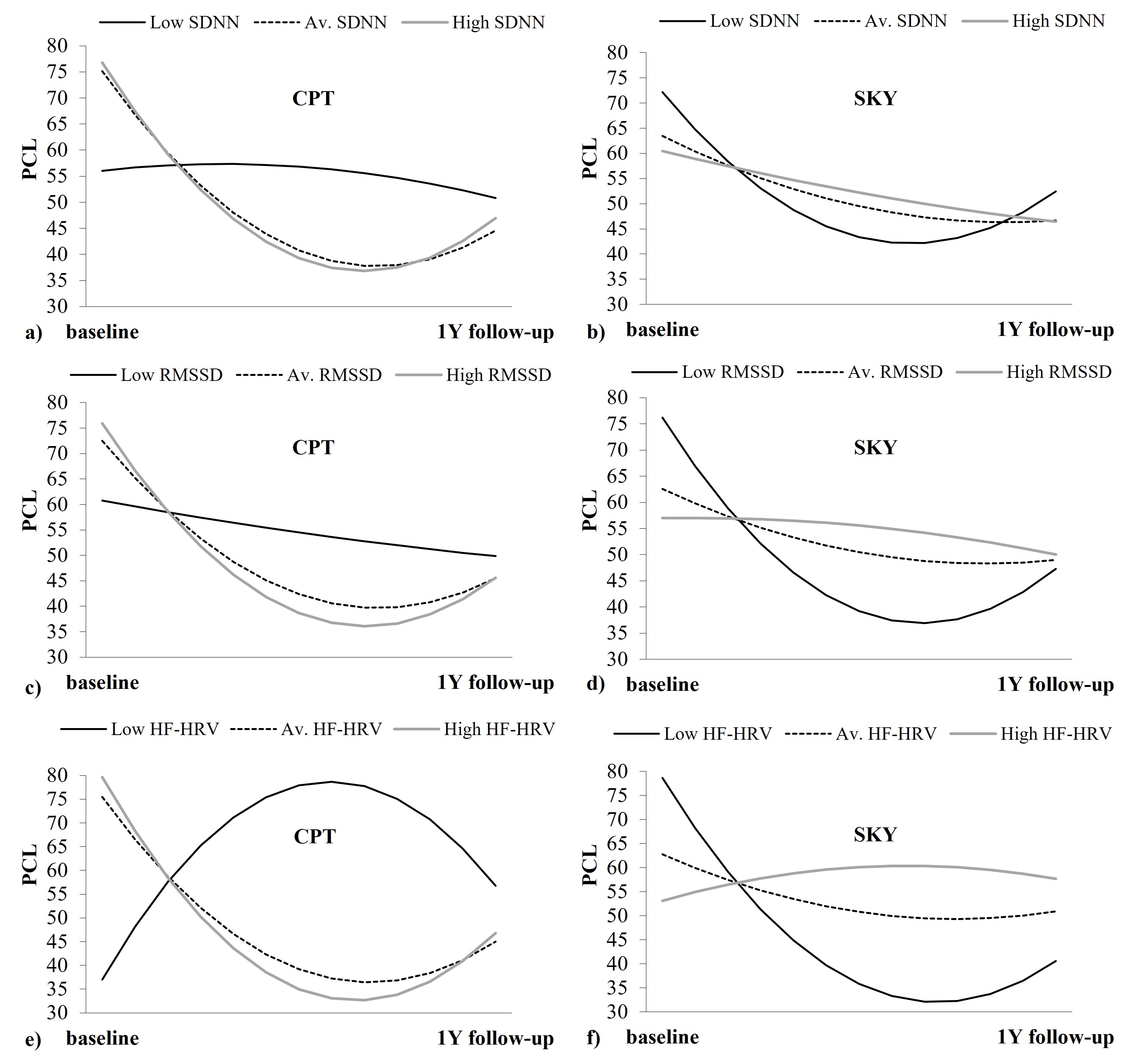

Supplement: Supplementary Figure 2 — Baseline HRV (where “low”/”high” refer to ±1SD from the mean and “av.” refers to the mean) as moderators of PTSD outcome with (A, C, E; left side) CPT and (B, D, F; right side) SKY, over the long-term: (A, B; top) SDNN, (C, D; middle) RMSSD, (E, F; bottom) HF-HRV. [file Image_2.jpeg]
